# Supplementary material for: Prevalence and associated factors of impaired renal function and albuminuria among adult patients admitted to a hospital in Northeast Ethiopia
Source: PLoS One. 2021 Feb 4;16(2):e0246509. doi: 10.1371/journal.pone.0246509 (PMC7861367; doi:10.1371/journal.pone.0246509)
Supplement: S1 File — (DOCX) [file pone.0246509.s001.docx]

**Questionnaire for a study on prevalence and associated factors of impaired renal function and albuminuria among adult patients admitted to a hospital in Northeast Ethiopia, 2020.**

| Identification No.__________________  PART 1- Questions related to socio-demographic characteristics | | | | |
| --- | --- | --- | --- | --- |
| \| Q. \|  \|  \| \| --- \| --- \| --- \| | QUESTION | | Check the Appropriate Option or Enter Numbers | |
| 100 | What is the sex of the respondent? | | 1. Male  2. Female | |
| 101 | What is the age of the respondent? | | Age in years (18-99) ________ (yy.m)  (write “99+”, if >99 years) | |
| 102 | Residence of the respondent | | 1. Urban  2. Rural | |
| 106 | What is the educational status of the respondent? | | \| 1. Illiterate/no formal  2. Grade1-8 \| 3. Grade9-12  4. College & above \| \| --- \| --- \| | |
| PART 2: Questions related to lifestyle habits, family history and awareness of kidney disease | | | | |
| 201 | Have you ever smoke cigarette in the past 12 month? | | 1.Yes  2. No | |
| 202 | Family History of kidney disease | | 1.Yes  2. No | |
| 203 | Have you been previously informed by a doctor or other health care provider that you have a failing kidney or kidney disease (excluding kidney stones, bladder infections, or incontinence)? | | 1.Yes  2. No | |
| PART 3: Clinical and Laboratory Measurements | | | | |
| 300 | Main clinical diagnosis of inpatient admission  (Comorbid condition for admission) | 1. Diabetes mellitus  2. Hypertension  3. Cardiovascular diseases (including coronary artery disease, myocardial infarction, heart failure, peripheral vascular disease, and old stroke)  4. Disease of the respiratory  5. HIV/AIDS  6. Others (specify)_________ | | |
| 301 | Current blood pressure measurement (Systolic/Diastolic) | 1^st^.________ mmHg  2^nd^. ________ mmHg  3^rd^.________ mmHg  Average_________ mmHg | | |
|  | Serum creatinine level at admission | ___________mg/dl | | |
| 305 | Serum creatinine level | ___________mg/dl | | |
|  | Urine dipstick |  | | |
|  | Albumin | 1.Negative  2. Positive | | \| Trace \| +1 \| +2 \| +3 \| \| --- \| --- \| --- \| --- \| |
|  | Blood | 1. Negative  2. Positive | | |
|  | Leucocytes | 1. Negative  2. Positive | | |
|  | Nitrate | 1. Negative  2. Positive | | |
|  | Glucose | 1. Negative  2. Positive | | |
|  | pH >8.0 | 1. Yes  2. No | | |
|  | Specific gravity >1.015 | 1. Yes  2. No | | |
